# General Practitioners Can Evaluate the Material, Social and Health Dimensions of Patient Social Status

**DOI:** 10.1371/journal.pone.0084828

**Published:** 2014-01-15

**Authors:** Sophia Chatelard, Patrick Bodenmann, Paul Vaucher, Lilli Herzig, Thomas Bischoff, Bernard Burnand

**Affiliations:** 1 Department of General Practice, University Joseph Fourier, Grenoble, France; 2 Vulnerable Population Unit, Department of Ambulatory Care and Community Medicine, University of Lausanne, Lausanne, Switzerland; 3 Department of Community Medicine and Primary Care, Geneva University Hospitals, Geneva, Switzerland; 4 Institute of General Practice, University of Lausanne, Lausanne, Switzerland; 5 Institute of Social and Preventive Medicine (IUMSP), Lausanne University Hospital, Lausanne, Switzerland; Universitat Rovira i Virgili, Spain

## Abstract

**Objective:**

To identify which physician and patient characteristics are associated with physicians' estimation of their patient social status.

**Design:**

Cross-sectional multicentric survey.

**Setting:**

Fourty-seven primary care private offices in Western Switzerland.

**Participants:**

Random sample of 2030 patients ≥16, who encountered a general practitioner (GP) between September 2010 and February 2011.

**Main measures:**

Primary outcome: patient social status perceived by GPs, using the MacArthur Scale of Subjective Social Status, ranging from the bottom (0) to the top (10) of the social scale.Secondary outcome: Difference between GP's evaluation and patient's own evaluation of their social status. Potential patient correlates: material and social deprivation using the DiPCare-Q, health status using the EQ-5D, sources of income, and level of education. GP characteristics: opinion regarding patients' deprivation and its influence on health and care.

**Results:**

To evaluate patient social status, GPs considered the material, social, and health aspects of deprivation, along with education level, and amount and type of income. GPs declaring a frequent reflexive consideration of their own prejudice towards deprived patients, gave a higher estimation of patients' social status (+1.0, p = 0.002). Choosing a less costly treatment for deprived patients was associated with a lower estimation (−0.7, p = 0.002). GP's evaluation of patient social status was 0.5 point higher than the patient's own estimate (p<0.0001).

**Conclusions:**

GPs can perceive the various dimensions of patient social status, although heterogeneously, according partly to their own characteristics. Compared to patients' own evaluation, GPs overestimate patient social status.

## Introduction

Health status and social status are linked along a social gradient from the top to the bottom of the social scale.[1] Patients' occupation, educational level, or income are often used as proxies for socioeconomic status.[2] More rarely a dedicated questionnaire is used to obtain a deprivation score.[3], [4] Social inequalities in health are found worldwide and have consequences for morbidity and mortality.[5] They have been studied in literature reviews regarding conditions like cancer[6] or heart failure.[7]

To mitigate the consequences of social inequalities on health, health care professionals, and GPs in particular, should detect patients suffering from those social inequalities. To reduce the consequences of deprivation on patients' health, GPs need to spend more time with deprived patient to build an empathic and open physician-patient relationship and to collaborate with other professionals.[8], [9] To implement these strategies, GPs need therefore to know their patient social status, and to recognize deprived patients. However, little is known about how GPs perceive deprivation. Several studies focussed more on its influence on handling care management.[10], [11] Regarding the actual perception of social status, a few qualitative studies[12]–[14] suggest that GPs take the social and material dimensions of deprivation into account and that they perceive their consequences in terms of access to health care, patient compliance, or psychological distress. But these issues are neither systematically nor explicitly covered during consultation, even though patients wish it would be.[12] To our knowledge, no attempt has been made so far to characterize how physicians perceive their patient social status in a quantitative study. In this context, the main objective of our study was to assess which GP and patient characteristics are associated with GPs' estimation of their patient social status. The second objective was to highlight and explain possible differences in estimations between GPs and patients.

## Methods

### Study design and participants

This survey was integrated in a cross-sectional multicentric study designed to investigate deprivation among patients visiting a convenience sample of GPs, working in urban and rural private practices, in Western French-speaking Switzerland. Each GP had to recruit up to fifty patients, randomly selected among all scheduled visits (one per half working-day). Data were collected from September 2010 to April 2011. Patients' inclusion criteria were: attending a GP during a day visit to the practice, age ≥16, ability to understand one national language, either French, German or Italian, or English, and informed consent. Recruitment ceased once physicians had included 50 patients or after 12 weeks.

### Ethics statement

The study was approved by the Ethical Committee of the Canton of Vaud under reference number 157/10. The Ethical Committee considered that requesting written consent was not necessary for this survey. Participants were informed both orally and by writing, that by filling in the questionnaire, they would thus provide their consent to participate. Given the study was exempted from any risk, it was considered that minors from 16 to 18 were capable of discernment and could give their consent without parental approval.

### Outcomes

**Figure 1:**
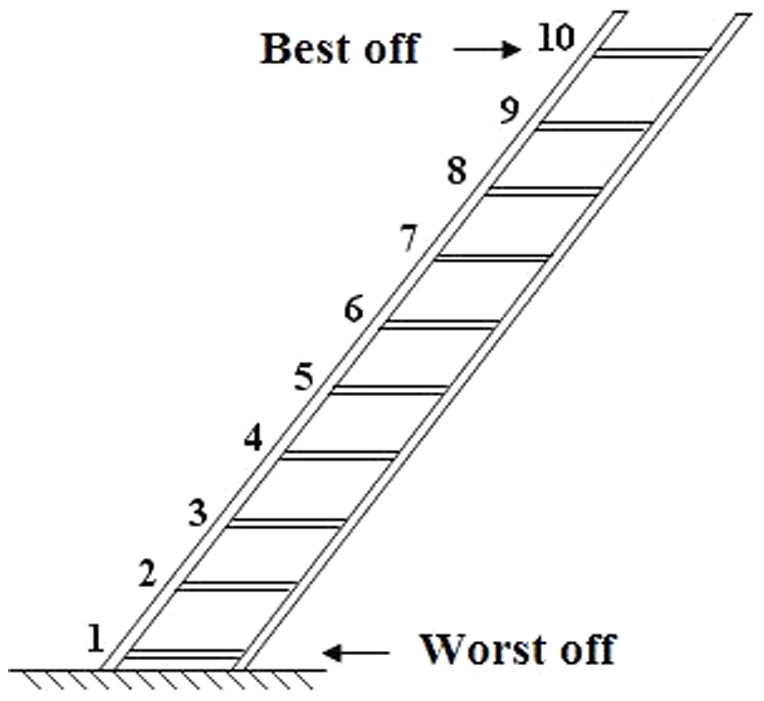
MacArthur scale of subjective social status. It is used to assess patient and doctor subjective evaluation of patient social status.

The primary dependent variable was measured by asking GPs to place each recruited patient on the validated MacArthur 10-step self-anchoring social status scale represented by a ladder graduated from 1 to 10 (**Figure 1**).[15] GPs were blinded to patient's answers to the self-administered questionnaire to be filled out in the waiting room. This patient questionnaire investigated material and social determinants of state-of-deprivation, self-perception of social status,[16] and state of health, as well as known socio-economic determinants of health as listed in **Table 1**. If necessary, the research staff followed up missing data with patients by phone. The DipCare score used to assess state-of-deprivation has been previously described and validated elsewhere.[4] It contains 16 questions exploring material, social and health deprivation. Self-perceived state of health was assessed through a visual analogue scale (VAS) from 0 to 100, as part of the validated Eq-5d questionnaire.[17] Following the recruitment period, a questionnaire was sent by post mail to all participating GPs. It contained questions about their general point of view regarding patients' deprivation and its influence on health and care, in addition to general socio-demographic information, as listed in **Table 2**.

**Table 1:** Potential patient level correlates of GPs' perception of patient social status.

Sex
Age
Educational level
Nationality*
Size and composition of household (presence of a spouse, number of children, other persons living in the household)
Amount of monthly income in household**
Source of income (wage, unemployment benefit etc.)***
Consultation length
Self-reported state of health: Visual analogue scale (VAS) of the EQ-5D questionnaire (0 to 100)
Deprivation: DipCare index (0 to 5) containing material (0 to 8), social (0 to 3) and health (0 to 5) deprivation sub-index
Self-perception of social position: MacArthur self-anchoring scale (1 to 10)

dichotomized to facilitate interpretation.

transformed into individual weighted monthly income, drawing its inspiration from OECD's modified equivalence scale.[17] The formula was:

Individual weighted income  =  Household total income/[(1+partner+x*teen+y*child)/(1+0.5*partner+0.5*x*teen+0.3*z*child)].

merged into two groups, reflecting either “stability” (wage and/or self-employed salary and/or retirement pension and/or assets) or “instability” of income (invalid's insurance pension and/or unemployment benefit and/or social welfare and/or loss-of-income insurance and/or alimony and/or study grant and/or family).

**Table 2:** Potential GP level correlates of GP's perception of patient social status*.

Sex
Age
Years in practice
Number of daily consultations
Proportion of deprived patients estimated by GP
Attention given to deprivation**
Feelings when taking in charge deprived patients**
Frequency of reflexive consideration of GPs' own prejudice towards deprived patients **
Influence that deprivation has on health and on care management, according to GPs**

See **Table 4** for detailed items.

dichotomized to facilitate interpretation.

**Table 2.** Potential GP level correlates of GP's perception of patient social status*.

### Statistical analysis

We used descriptive univariable statistics and a two-level random effect model considering patients clustered in general practices. We purposely choosed this approach, because it allowed us to take heterogeneity between GPs into account; and to explain GPs' perception of social status with both patient and GP characteristics.[18] As we used linear regression for a measure that by nature is not a real number, linearity of association was tested and variables were transformed if the association was not linear.We first tested a non-adjusted model, only taking into account the cluster effect but no explanatory variable, to highlight a possible inter-group heterogeneity. Then we tested every patient's variable in a univariable mixed model as predictor of the random intercept. We chose to test all of them, as they are all known to be determinants of socioeconomic status.[2], [19] The next step was to test each GP variable as predictor of random intercept. We did not have any literature-based hypothesis to suggest which GP characteristics would interfere with the dependent variable, so we tested them one by one in an exploratory way. We eliminated all variables with P values above 0.20. We then built a multivariable model using a step-down regression, adjusted first for patient characteristics, and finally fully adjusted for patient and GP characteristics. For each model, we calculated the intraclass correlation coefficient (ICC) that represents the concordance between the different GPs in evaluating patient social status. To fulfill the secondary objective, we undertook another series of analyses following the same steps, but with the difference between GP's and patient's evaluation as the dependent variable. We used Stata version 12.0 for analyses.

## Results

### Patient and physician socio-demographic characteristics

Forty seven out of 50 GPs accepted to participate (94%). Among the 2600 patients considered for eligibility, 2030 were recruited (78%). **Table 3** shows patient characteristics. We compared the 297 (14.8%) patients who did not give their monthly income with the respondents **(Table S1)**. Individuals who did not answer about income amount were more often women, non-Swiss, older, and their social deprivation index was higher. **Table 4** shows GP characteristics. Forty six among the 47 GPs answered the questionnaire.

**Table 3:** Characteristics of the 2030 patients.

VARIABLES			n	Mean (+/− SD) or Percentage (raw number)	[10th and 90^th^ percentile]
**Sex(Male)**			1994	41% (818)	
**Age (Years)**			1987	55.3 (+/− 18.1)	[28 – 79]
**Education level**	Incomplete compulsory schooling		1943	5.1% (99)	
	Complete compulsory schooling			22.5% (437)	
	General and vocational training			48.9% (950)	
	Higher education			23.5% (457)	
**Nationality (more than one possible answer)**	Swiss		2030	80% (1623)	
	European			21.8% (442)	
	Other			3.5% (70)	
**Presence of a spouse**			1976	61% (1202)	
**Number of children in the household**		0	1973	60% (1187)	
		1		14% (274)	
		2		18% (355)	
		>2		8% (157)	
**Monthly household income (SFr)**			1684	6648 (+/− 5836)	[2300 – 12000]
**Monthly individual income (SFr)**			1684	3258 (+/− 3388)	[1200 – 5750]
**Monthly individual weighted income (SFr)** *			1684	4827 (+/− 4241)	[2055 – 7875]
**Sources of income (more than one possible answer)**	Wage		2030	50.5% (1026)	
	Retirement pension			35.7% (724)	
	Invalid's insurance pension			9.1% (185)	
	Assets (property, shares)			8.0% (163)	
	Unemployment benefit/Social welfare			7.4% (151)	
	Self-employed salary			7.1% (144)	
	Widow's pension/Alimony			6.2% (126)	
	Parents/family/friends			4.4% (90)	
	Loss-of-income insurance			2.5% (50)	
	Study grant			0.8% (17)	
**Consultation length (minutes)**			1997	23.6 (+/− 9.9)	[15 – 35]
**VAS Eq5d score** ** **(out of 100)**			1963	68.4 (+/− 19.5)	[40 – 90]
**DipCare social deprivation index (0 to 5)**			1987	1.6 (+/− 1.5)	[0 – 4]
**DipCare material deprivation index (0 to 8)**			2001	1.1 (+/− 1.8)	[0 – 4]
**DipCare health deprivation index (0 to 3)**			1994	0.4 (+/− 0.7)	[0 – 2]
**DipCare global deprivation index (0 to 5)**			1938	1.24 (+/− 1.24)	[0 – 3]
**Patient MacArthur scale (0 to 10)**			1978/	5.9 (+/− 1.8)/	[4]–[8]
**GP MacArthur scale (0 to 10)**			2007	6.3 (+/− 2.1)	[3]–[9]
**Difference of evaluation**			1957	0.5 (+/− 2.1)	[−2 – 3]

SD: standard deviation.

Poverty threshold in Switzerland (2010): 2250 SFr for a single person, and 4000 SFr for a couple with 2 children.

Self-perceived state of health assessed through a visual analogue scale (VAS) from 0 to 100, as part of the validated Eq-5d questionnaire.

**Table 4:** GP characteristics.

VARIABLES		n	Mean (+/−SD) or Percentage		[10th and 90^th^ percentile]
**Sex (Male)**		47	72.3%		
**Age (Years)**		44	54 (+/− 9)	[39 – 63]	
**Years of practice**		44	18.9 (+/− 10.6)	[2 – 30]	
**Place of practice**	Urban	44	31.8%		
	Rural		31.8%		
	Suburbs		36.4%		
**Number of daily consultations**		47	19.9 (+/− 7.7)	[10 – 28]	
**Proportion of deprived patients**	<10%	46	18.2%		
	10–20%		29.6%		
	20–30%		47.7%		
	30–40%		4.5%		
Attention given to deprivation*	Average	45	57.8%		
	Much		42.2%		
**Consultation planning with deprived patients**	Less time	43	2.3%		
	Same time		53.5%		
	More time		44.2%		

		**n**	**Not at all**	**Somewhat**	**Pretty much**	**Very much**
**Feelings when taking care of deprived patients**	Gratification/Self-righteousness	44	18.2%	29.6%	47.7%	4.5%
	Frustration	46	19.6%	30.4%	37%	13.0%
	Overwork	46	30.4%	39.1%	19.6%	10.9%
	Powerlessness	46	4.3%	28.3%	32.6%	34.8%
	Normal role for a doctor	45	8.9%	17.8%	37.8%	35.5%
	Take all the misery of the world	45	48.9%	35.6%	15.5%	0
			**No**	**Probably not**	**Probably yes**	**Yes**
**Thinking that patients wish to talk about deprivation issues**		46	2.2%	28.9%	48.9%	20.0%
			**No**	**Rarely**	**Sometimes**	**Often**
**Reflexive consideration on own prejudice towards deprived patients**		46	0%	13.3%	46.7%	40.0%
			**Not at all**	**Somewhat**	**Prettymuch**	**Verymuch**
**Influence of deprivation on**	General care management		13.1%	23.9%	23.9%	39.1%
	Doctor-patient relationship	46	23.9%	30.4%	37%	8.7%
			**No**	**Rarely**	**Sometimes**	**Often**
**Influence of deprivation on care management**	Choice of a less costly treatment	46	26.1%	15.2%	28.3%	30.4%
	Less medical investigations		39.1%	19.6%	28.3%	13%
	Question about difficulties to pay		8.7%	13%	32.6%	45.7%

SD: standard deviation *No answers for categories «none» and «little».

### Primary outcome multilevel analysis

In univariable analysis (full analysis in **Table S2)**, almost all variables at patient level were significantly associated with GP's evaluation of patient social status (P value of 0.20 used in the variable selection process). When the DipCare deprivation index increased in any of its three dimensions, the social status score was lower. Stability of income was related to a higher score whereas instability was linked to a lower score, without a strong correlation between these 2 variables (r  = −0.48).

Regarding GP characteristics in univariable analysis, years of experience, feelings of gratification, overwork, and powerlessness were associated with higher subjective score, as well as thinking that patients wish to talk about deprivation issues and having reflexive consideration of their own prejudice towards deprived patients. On the contrary, thinking that deprivation has an influence on care management and applying these changes (less costly treatment, fewer investigations, and asking questions about material difficulties) was associated with lower scores.

Non-adjusted and final multivariable models are shown in **Table 5**. We chose to keep the weighted income as it had the biggest coefficient in univariable analysis and corresponds more to reality. Both the DipCare health deprivation index and the EQ-5D explore the health dimension and had comparable univariable coefficients. We used the DipCare health deprivation index, because we wanted to keep the coherence of the DipCare global deprivation index in its three dimensions. Among patient characteristics, gender, nationality and consultation length were excluded from the model. Two GP characteristics were included in the final model. Reflective consideration of GPs' own prejudice towards deprived patients increased the score by broadly 1 point. Prescribing cheaper treatment to deprived patients decreased the score by 0.75 point. In the final model, the intraclass correlation coefficient was 0.20 (compared to 0.21 in the non-adjusted model). That shows the degree of remaining heterogeneity between physicians, after taking some of their personal characteristics into account.

**Table 5:** Multivariable analysis of GP's evaluation of patient social status.

		Non-adjusted model	Univariable model		Final multivariable model (n = 1519)	
			Unadjusted coeff	p-value	Coeff	p-value
Constant		**6.329**			**4.894**	
**FIXED EFFECT – VARIABLES AT PATIENT LEVEL**						
**Age**			0.016	0.000	0.019	0.000
**Educational level** (Ref: Incomplete compulsory schooling)	Complete compulsory schooling		0.690	0.000	0.474	0.013
	General and vocational training		1.279	0.000	0.656	0.000
	Higher education		2.246	0.000	1.263	0.000
**Presence of a spouse**			0.825	0.000	0.378	0.000
**Number of children**			0.104	0.006	0.083	0.016
**Monthly individual weighted income (by 1000 SFR)**			0.128	0.000	0.044	0.000
**Unstable income (composite)**			−1.548	0.000	−0.577	0.000
**Stable income (composite)**			1.825	0.000	0.574	0.000
**Social deprivation index (0 to 5)**			−0.422	0.000	−0.266	0.000
**Material deprivation index (0 to 8)**			−0.429	0.000	−0.128	0.000
**Health deprivation index (0 to 3)**			−0.709	0.000	−0.200	0.001
**FIXED EFFECT – VARIABLES AT DOCTOR LEVEL**						
**Attention given to prejudice and stereotype regarding deprivation** §			0.809	0.053	1.034	0.002
**Influence of deprivation on patient's management** §	Choice of a less costly treatment		−0.846	0.002	−0.744	0.002
**RANDOM EFFECT**						
**Variance (√) at doctor level**		0.931			0.723	
**Residual variance (√) at patient level**		1.813			1.435	
**Intraclass correlation coefficient**		0.21			0.20	

Dichotomized variables (0 = No or Rarely; 1 = Sometimes or Often).

(√)  =  square root.

### Secondary outcome

On average, GPs gave a higher score to patients' social status than patients themselves. The mean difference between GP's and patient's subjective evaluation was 0.5 ([−0.54; −0.36], p<0.0001). Pearson's correlation coefficient between the two evaluations was 0.43. **Table 6** presents the modelling of this difference of evaluations. GPs «overestimated» patient social status (compared to patient's own evaluation) to a greater extent when patient educational level increased, or when patients benefited from a “stable” income. When GPs said they reflexively considered their own prejudice regarding deprived patients, they gave a higher score than patients themselves. On the contrary, when GPs declared that they adapted their care management by giving cheaper treatment to deprived patients, this “overestimation” was reduced.

**Table 6:** Multivariable analysis of the difference between GP and patient evaluation of patient social status.

			Non-adjusted model	Univariable model		Final multivariable model (n = 1732)	
				Unadjusted coefficients	p-value	Coefficient	p-value
Constant			**0.464**			**3.713**	
**FIXED EFFECT – VARIABLES AT PATIENT LEVEL**							
**Patient's self evaluation**				−0.547	0.000	−0.798	0.000
**Patient's age**				0.010	0.000	0.016	0.000
**Educational level** (Ref: Incomplete compulsory schooling)	Complete compulsory schooling			0.551	0.011	0.538	0.002
	General and vocational training			0.630	0.002	0.752	0.000
	Higher education			0.626	0.004	1.292	0.000
**Presence of a spouse**				0.304	0.001	0.391	0.000
**Unstable income (composite)**				−0.523	0.000	−0.469	0.000
**Stable income (composite)**				0.729	0.000	0.675	0.000
**Material deprivation index (0 to 8)**				−0.051	0.041	−0.093	0.000
**Health deprivation index (0 to 3)**				−0.081	0.184	−0.140	0.009
**FIXED EFFECT – VARIABLES AT DOCTOR LEVEL**							
**Attention given to prejudice and stereotype regarding deprivation** §				0.882	0.015	0.978	0.004
**Influence of deprivation on patient's management** §		Choice of a less costly treatment		−0.638	0.009	−0.753	0.002
**RANDOM EFFECT**							
**Variance (√) at doctor level**			0.819			0.740	
**Residual variance (√) at patient level**			1.890			1.432	
**Intraclass correlation coefficient**			0.16			0.21	

Dichotomized variables (0 = No or Rarely; 1 = Sometimes or Often)(√)  =  square root.

## Discussion

In our study, GPs estimate patient social status by taking into account three aspects of deprivation – material, social, and health status – using specifically educational level and type and amount of income. Furthermore, the frequency with which GPs consider their own prejudice towards deprived patients, and the way they actually adapt their care management for such patients are also linked with their estimation of patients' social status. On average, GPs estimate patient social status to be higher than do the patients themselves.

### Limitations

GPs included in this study were conveniently selected. Their interest in this research study may mean that they were particularly aware of social inequalities in health. Our findings may then not be extrapolated to all GPs. However they were spread across all urban and rural areas in Western Switzerland, which corresponds to one quarter of the country population. Almost 300 patients did not declare their income amount; they did not differ from the other patients regarding the dependent variable. We tested our final model without income, in order to reintegrate these 300 patients into the analysis. It did not change the magnitude of other variable coefficient (results not shown). In addition, because of the comparatively large number of patients and small number of GPs, we may have missed important GP characteristics and included irrelevant patient characteristics in our statistical analysis.

### Strengths

The main strengths of our study are the randomized selection of patients, and the large number of participating GPs and patients. To our knowledge, our work is original, as it studied for the first time physicians' perception of their patient social status in a quantitative way. In addition, all dimensions of deprivation were assessed using a validated score. A multilevel model allowed us to add some new perspectives by accounting for GP heterogeneity and GP characteristics. Hierarchical analysis is often used to study social inequalities in health, mostly to characterize the effect of community or geographical context (i.e., neighbourhood's social position) on patient's health.[20] Here patients were nested in general practices, which allowed us to highlight some heterogeneity between GPs, and the role their characteristics play in modifying their evaluation. In this way, the intraclass correlation coefficient can be used as a potential source of information and not just an adjustment for nuisance.

### Comparison with literature

Our quantitative findings support results from previous qualitative studies, which show that GPs are able to perceive the material dimension as well as social support and state of health, including psychological health, to get an insight into patient social status.[14] In this way, our results slightly differ from Bloch's,[13] who interviewed Canadian experts on poverty and health, about how primary care professionals took care of these issues. They pointed out the lack of understanding of professionals about the reality of deprivation and its consequences. Indeed, nearly one out of five GPs in our study never or rarely asked patients if they had difficulties with consultation cost, and one out of ten thought it was not the GP's role to take care of deprivation issues. However, most of them acknowledge the influence of socioeconomic factors on health. Barry[12] studied patients' agenda and if physicians actually met this agenda during the consultation. He showed that although social context is a subject of interest for patients, it is often neglected by physicians. In our study, about one third of GPs believed that patients do not wish to talk about deprivation issues.

### Interpretation and implications for future research

Our results show that GPs who were trying to consider deprived patients without prejudice seemed to “overestimate” patient social status. Our hypothesis to explain this is that they tried, in a reflective way, not to stigmatise deprived patients. Furthermore they may be rather optimistic about patients' own resources other than material or given by social support. Thus, this “overestimation” would not mean that GPs did not understand their patients' context, but that they tended to “see the glass half-full”. On the other hand, we found that GPs who were more active and adapted their care management depending on patient social status tended to “underestimate” this status. It seems that the type of income did matter for GPs and explained part of the difference between GP's and patient's evaluations. Therefore we suggest that GPs who adapt care management give more importance to financial issues than patients actually do.

Over one half of GPs in our study combined two approaches that we can call “reflexive” and “active”, so that the two coefficients almost cancel each other out, reducing the difference between both evaluations. Indeed, GPs' ability to consider their own prejudice regarding deprived patients and to adapt their management when needed seemed to match patient's own evaluation of their situation.

The current study was designed to answer a complex question. The way social status is perceived depends on both objective data and GP's subjectivity. In daily practice, GPs do not usually have access to all the information derived from our patient questionnaire (i.e., exact amount of income, nature of social support, housing salubrity etc.). Actually, the model explaining GP evaluation contains variables that are not always known to the GP. On the contrary, GPs, thanks to long term follow-up they have of many patients, may know other facts that may change their perception (i.e., traumatic life events, working conditions, family context etc.). This complexity may have contributed to the large part of unexplained variance in our models. Further studies on this topic are recommended to confirm our results, ideally with a random sample of physicians.

It has been shown that patients from lower social classes receive less information, with a more directive and a less participatory consulting style from their doctor than other patients do.[10] In hospital setting, physicians gave lower socioeconomic status (SES) patients more negative ratings on personality characteristics (lack of self-control, irrationality) and level of intelligence. In addition, lower SES patients were rated as less likely to be compliant.[11] Additional exploration should focus on the real process leading physicians to adapt their practice depending on the actual perception they have of their patient social status. The notion of “social concordance” between patient and physician seems to play a great role in this process.[21]

In future investigations, it would also be interesting to evaluate an intervention consisting in encouraging physicians to ask questions about the social context. Then we could evaluate if their subjective evaluations change and tend to meet the patients' ones.

The final goal of determining patients social status is to enhance patients health. We strongly hypothesize that a better knowledge of patient social context leads to better global care, only if physicians use this knowledge to go beyond their own bias.

## Conclusions

GPs can evaluate patient social status in its various dimensions, with a tendency to overestimation, compared to patient's own evaluation. The way GPs consider deprivation, in both reflexive and active ways, influences their evaluation, and is partly responsible for the difference between GP's and patient's evaluation.

## Supporting Information

Table S1 **Comparison between patients who have or have not answered about their household's income amount, using chi-square and t-test.**
(DOCX)

Table S2 **Univariable analysis of GP evaluation of patient social status, with patient and doctor level variables.**
(DOCX)
