# Supplementary material for: General Practitioners Can Evaluate the Material, Social and Health Dimensions of Patient Social Status
Source: PLoS One. 2014 Jan 15;9(1):e84828. doi: 10.1371/journal.pone.0084828 (PMC3893170; doi:10.1371/journal.pone.0084828)
Supplement: Table S1 — Comparison between patients who have or have not answered about their household's income amount, using chi-square and t-test. (DOCX) [file pone.0084828.s001.docx]

**Table S1: Comparison between patients who have or have not answered about their household’s income amount, using chi-square and t-test**

| **VARIABLES** | **n** | **Income given**  **n=1733** | **Income not given**  **n=297** | **p-value** |
| --- | --- | --- | --- | --- |
| **Sex (women)** | 1994 | 57.7% | 66.5% | 0.005 ** |
| **Age (years)** | 1987 | 54.8 | 58.3 | 0.004 ** |
| **Educational level (>compulsory)** | 1943 | 72.9% | 69.1% | 0.33 |
| **Nationality (Swiss)** | 2033 | 80.7% | 75% | 0.024 ** |
| **Presence of a spouse** | 1976 | 60.7% | 61.6% | 0.78 |
| **Unstable income** | 2033 | 22.4% | 23.3% | 0.72 |
| **Consultation length (minutes)** | 1997 | 23.5 | 24 | 0.45 |
| **VAS Eq5d score** | 1963 | 68.7 | 66.2 | 0.053 |
| **Social deprivation index** | 1987 | 1.59 | 1.86 | 0.005 ** |
| **Material deprivation index** | 2001 | 1.12 | 1.05 | 0.55 |
| **Health deprivation index** | 1994 | 0.44 | 0.43 | 0.81 |
| **Patient’s MacArthur scale (0 to 10)** | 1978 | 5.86 | 5.99 | 0.28 |
| **Doctor’s MacArthur scale (0 to 10)** | 2007 | 6.30 | 6.49 | 0.14 |
| **Difference of evaluation** | 1957 | 0.43 | 0.55 | 0.39 |

** p<0.05
