# Supplementary material for: General Practitioners Can Evaluate the Material, Social and Health Dimensions of Patient Social Status
Source: PLoS One. 2014 Jan 15;9(1):e84828. doi: 10.1371/journal.pone.0084828 (PMC3893170; doi:10.1371/journal.pone.0084828)
Supplement: Table S2 — Univariable analysis of GP evaluation of patient social status, with patient and doctor level variables. (DOCX) [file pone.0084828.s002.docx]

**Table S2: Univariable analysis of GP evaluation of patient social status, with patient and doctor level variables**

| **VARIABLES AT PATIENT LEVEL** | | | **n** | **Coefficient** | **p-value** |
| --- | --- | --- | --- | --- | --- |
| **Sex (Male)** | | | 1974 | 0.261 | 0.002** |
| **Age (Years)** | | | 1967 | 0.016 | 0.000** |
| **Educational level** (Ref: Incomplete compulsory schooling) | | Complete compulsory schooling | 1943 | 0.690 | 0.000** |
|  | | General and vocational training |  | 1.279 | 0.000** |
|  | | Higher education |  | 2.246 | 0.000** |
| **Nationality (Non-Swiss)** | | | 2007 | 0.562 | 0.000** |
| **Presence of a spouse** | | | 1955 | 0.825 | 0.000** |
| **Number of children in the household** | | | 1953 | 0.104 | 0.006** |
| **Monthly household income (by 1000 SFr)** | | | 1712 | 0.102 | 0.000** |
| **Monthly individual income (by 1000 SFr)** | | | 1664 | 0.105 | 0.000** |
| **Monthly individual weighted income (by 1000 SFr)** | | | 1664 | 0.128 | 0.000** |
| **Sources of income** | Wage | | 2006 | 0.265 | 0.001** |
|  | Self-employed salary | |  | 0.462 | 0.004** |
|  | Retirement pension | |  | 0.601 | 0.000** |
|  | Invalid’s insurance pension | |  | -1.494 | 0.000** |
|  | Unemployment benefit | |  | -1.439 | 0.000** |
|  | Social welfare | |  | -2.119 | 0.000** |
|  | Loss-of-income insurance | |  | -0.575 | 0.030** |
|  | Widow’s pension | |  | 0.013 | 0.951 |
|  | Alimony (divorce) | |  | -1.188 | 0.000** |
|  | Study grant | |  | -0.920 | 0.039** |
|  | Assets (property, shares) | |  | 1.375 | 0.000** |
|  | Parents / family / friends | |  | -0.317 | 0.109* |
| **Unstable income (composite)** | | | 2006 | -1.548 | 0.000** |
| **Stable income (composite)** | | | 2006 | 1.825 | 0.000** |
| **Consultation length (minutes)** | | | 1989 | -0.007 | 0.145* |
| **VAS Eq5d score in 3 categories** (Ref : VAS >80) | <50 | | 2007 | -0.417 | 0.000** |
|  | 50-80 | |  | -1.350 | 0.000** |
| **Social deprivation index** | | | 1965 | -0.422 | 0.000** |
| **Material deprivation index** | | | 1978 | -0.429 | 0.000** |
| **Health deprivation index** | | | 1972 | -0.709 | 0.000** |
| **Global deprivation index** | | | 1918 | -0.650 | 0.000** |

* p<0.20  ** p<0.05

| **VARIABLES AT DOCTOR LEVEL** | | | | | **n** | **Coefficient** | **p-value** |
| --- | --- | --- | --- | --- | --- | --- | --- |
| **Sex (Man)** | | | | | 2007 | 0.047 | 0.883 |
| **Age (Years)** | | | | | 1906 | 0.018 | 0.257 |
| **Years of practice^§^** | | | | | 1906 | 0.506 | 0.074* |
| **Place of practice (Ref: Urban)** | | Rural | | | 1877 | 0.281 | 0.447 |
|  | | Suburbs | | |  | 0.108 | 0.761 |
| **Number of daily consultations (Ref: 20/29)** | | 0/19 | | | 2007 | 0.211 | 0.484 |
|  | | 30/50 | | |  | -0.500 | 0.388 |
| **Proportion of deprived patients (Ref: <10%)** | | 10-20% | | | 1972 | 0.218 | 0.520 |
|  | | 20-30% | | |  | 0.066 | 0.876 |
|  | | 30-40% | | |  | -0.030 | 0.976 |
| **Attention given to deprivation (Much)** | | | | | 1931 | 0.374 | 0.207 |
| **Feelings when taking care of deprived patients** | Gratification / Self-righteousness | | | | 2007 | 0.362 | 0.197* |
|  | Frustration | | | |  | 0.228 | 0.511 |
|  | Overwork | | | |  | 0.451 | 0.140* |
|  | Powerlessness | | | |  | 0.699 | 0.013** |
|  | Normal role for a doctor | | | |  | 0.485 | 0.252 |
|  | Take all the misery of the world | | | |  | 0.241 | 0.394 |
| **Positive feelings (composite)^§§^** | | | | | 2007 | 0.477 | 0.085* |
| **Negative feelings (composite)^§§^** | | | | | 2007 | 0.451 | 0.140* |
| **Patients want to talk about deprivation issues (Ref: No)** | | | | Probably not | 1955 | 0.741 | 0.435 |
|  | | | | Probably yes |  | 0.920 | 0.326 |
|  | | | | Yes |  | 1.576 | 0.102* |
| **Patients want to talk about deprivation issues^§§^** | | | | | 1955 | 0.748 | 0.032** |
| **Attention given to prejudice regarding deprivation (Ref : Rarely)** | | | | Sometimes | 1924 | 0.909 | 0.038** |
|  | | | | Often |  | 0.690 | 0.123* |
| **Attention given to prejudice regarding deprivation ^§§^** | | | | | 1924 | 0.809 | 0.053* |
| **Consultation planning with deprived patients**  (Ref: Same time) | | | | Less time | 1866 | 0.436 | 0.657 |
|  | | | | More time |  | 0.288 | 0.336 |
| **Influence of deprivation on** | | | Medical management^§§^ | | 1972 | -0.782 | 0.059* |
|  | | | Doctor-patient relationship ^§§^ | |  | 0.116 | 0.732 |
| **Influence of deprivation on patient’s management^§§^** | | | Choice of a less costly treatment | | 1972 | -0.846 | 0.002** |
|  | | | Less medical investigations | |  | -0.834 | 0.002** |
|  | | | Question about difficulties to pay | |  | -0.681 | 0.042** |

^§^ 20 years and less vs more than 20 years ^§§^ Dichotomized variables (0=No/Not at all or Somewhat/Rarely/Probably not ; 1=Probably yes/Pretty much/Sometimes or Yes/Very much/Often)  * p<0.20  ** p<0.05
